# Why, Immunologically, Housing‐Related Fungi and Endotoxins (and Other Chronic Pro‐Inflammatory Stressors) Risk Latent Tuberculosis Reactivation, Severe Asthma, and Translocating and Invasive Infections in Indigenous Communities in Canada

**DOI:** 10.1002/ajhb.70258

**Published:** 2026-04-13

**Authors:** Stacie Burke

**Affiliations:** ^1^ Department of Anthropology University of Manitoba Winnipeg Manitoba Canada

**Keywords:** immunity switches, Indigenous health, inflammation, mucosa, neutrophil, reactivation, tuberculosis

## Abstract

Type 1/M1/T_H_1 and type 3/M1/T_H_17 pro‐inflammatory switches are risks for latent *Mycobacterium tuberculosis* (Mtb) reactivation and ongoing infection transmission. This paper considers the heavy toll of reactivation risk in Indigenous communities in Canada and the chronic, everyday pro‐inflammatory stressors connected with type 3/M1/T_H_17 immune switching, including household fungal and endotoxin exposures that fuel reactivation risk. The paper argues that regular or chronic pro‐inflammatory stressors are risks not only for latent Mtb reactivation and ongoing transmission, but also, via compromised type 4/M2/T_H_22 mucosal barrier protections, severe asthma and translocated, possibly invasive, bacterial, viral, and fungal infections. Individual and community efforts to reduce chronic pro‐inflammatory stressors are important, but as the immunological, switch‐inducing rationale suggests, unless chronic pro‐inflammatory risks associated with housing are mediated, tuberculosis reactivation and other health risks will likely persist.

## 1 Why Is Nunavik in the News?

Nunavik occupies the northern third of the province of Québec, Canada, including 14 coastal villages home to Nunavimmiut.^1^ A recent CBC News (2025) article reported a “spike” in tuberculosis cases, “rates far above those typically seen in Québec and Canada.” With 83 cases of active tuberculosis identified by September 2025, Nunavik's incidence rate was on track for about 800 cases per 100 000 people, “making it among the highest in the world.” Globally, the Marshall Islands, the Philippines, and Myanmar report the highest incidence rates at 692, 643, and 558 per 100 000, respectively (WHO 2024). As of early December 2025, the number of active cases of tuberculosis in Nunavik had increased further to 112 (Wat 2025). Outside Nunavik's spike, Orr et al. (2024) report tuberculosis incidence rates of 136.7 and 21.4 per 100 000 in Inuit^2^ and First Nations communities, respectively, compared to 0.3 per 100 000 in the non‐Indigenous Canadian‐born population.^3^ In high incidence contexts such as Nunavik, large numbers of community members will have latent Mtb infection (sleeping TB).

## 2 Why Does Sleeping TB Require Vigilance?

As long as latent Mtb infections persist, so do risks for reactivation, new transmissions, and endemic and epidemic circulations. As Dunn et al. (2022) note, the “large pool of latent TB infection across all regions in Inuit Nunangat”^4^ presents as “a high risk for future outbreaks.” While reactivations typically occur in about 10% of latent Mtb cases, chronic and ingrained niche‐based^5^ Pro‐inflammatory stressors can push the scope for reactivation risk beyond that which is typically expected. A review of latent Mtb reactivation in Manitoba between 1976 and 1981 identified disproportionate risks of reactivation among First Nations people, contributing to ongoing tuberculosis transmission (Johnson et al. 1985). Likewise, Clark and Vynnycky's (2004) study of British Columbia First Nations found that between 1972 and 2000, reactivation risks were an estimated “threefold greater” than for other populations. In a 1971 study of reactivation among Inuit in Canada, Chan‐Yeung et al. (1971) noted “a high rate of reactivation,” with the emerging pulmonary disease often “extensive” and cavitary, two characteristics aligned with greater infectiousness and likelihood of transmission. Both Clark and Vynnycky (2004) and Lee et al. (2015) describe how a “high prevalence of latent infection” triangulates with ongoing tuberculosis transmission risk in Indigenous communities. In one Nunavik village, Lee et al. (2015) traced a single case of latent Mtb reactivation emerging in 2007 which led to secondary and tertiary transmissions such that, by 2011–2012, 50 new cases of active disease had been diagnosed in a population of 933. Tyler et al. (2017) argue unless “transmission chains of endemic TB” originating from latent Mtb reactivations are interrupted in Indigenous communities, tuberculosis will persist. Despite these risks, there is an “unacceptable data quality” in Canada with a lack of “population‐level data on the characteristics that increase the likelihood of reactivation of latent TB infection,” even basic counts of “the total number of individuals in Canada with latent TB infection” (Essue et al. 2018).

## 3 Why Is Anti‐Inflammatory Type 2/M2/T_H_2 Immunity Necessary to Keep Mtb Sleeping?

We are conditioned to think of “immunosuppression” as a risk for latent tuberculosis reactivation and disease, but reactivations are more likely to occur when tuberculosis bacteria take advantage of favorable pro‐inflammatory immunity switches to escape from intact and well‐functioning granulomas (Burke 2025). In the body, the four arms of immunity (type 1, type 2, type 3, and a type 4 protective arm) exist in careful balance, the pro‐inflammatory arms (type 1, type 3) and the anti‐inflammatory arms (type 2, type 4) counterbalanced and see‐sawing in relation to one another (Eberl 2016; Medzhitov 2008; Rankin and Artis 2018; Xia et al. 2023). As one of the four arms is engaged, Gérard Eberl (2016) describes, the other three are repressed. Immunity switches occur within an ongoing negotiation of balance, responding to stressors and then returning to homeostasis, with macrophage polarizations, the particular cytokine milieu, and the dominance of T helper cells also switching in turn (Eberl 2016; Rankin and Artis 2018). In primary *Mycobacterium tuberculosis* infection, a pro‐inflammatory switch is drawn to type 1 immunity, first engaging innate immunity's M1 polarized macrophages, followed by adaptive immunity's T_H_1 T helper cells and associated cytokines, notably IFN‐ƴ (interferon‐gamma), for a composite type 1/M1/T_H_1 inflammatory immune response. The cytokine array is important for intercellular communication and “orchestrating” the correct immune response to pathogens and tissue injuries (Valeri and Raffatellu 2016).

Following the pro‐inflammatory type 1 switch, virulent tuberculosis bacteria can draw upon diverse adaptations (virulence factors) to manipulate the human host's inflammatory response, sometimes driving an anti‐inflammatory type 2/M2/T_H_2 immune switch and then settling into granulomas. Granulomas are the host's attempt to keep the infection latent and contained, but Mtb remain vigilant for suitable reactivation opportunities, periodically awakening within their granulomas to “sample the immune environment,” reading the signs delivered to them hematogenously via the granuloma's vascular connections (Shaler et al. 2013). Permissive pro‐inflammatory switches to either type 1/M1/T_H_1 or type 3/M1/T_H_17 immunity induced by injuries or infections elsewhere in the body can be read as opportunities, and, in response, well‐adapted Mtb can drive the repolarization of their macrophages to a VEGFA‐expressing M1 phenotype and, via angiogenesis (the sprouting of new blood vessels), create a vascular escape route from otherwise intact and well‐functioning granulomas (Burke 2025; Oehlers et al. 2015; Polena et al. 2016). Because latent Mtb reactivations are risks for cycles of endemicity or epidemic outbreaks, it is critical to identify niche‐based stressors which, in addition to injuries and infection, drive pro‐inflammatory switches, situating immunological risks for latent Mtb reactivation in community context.

Classically, intracellular bacterial and viral infections draw type 1/M1/T_H_1 immune switches, while type 3/M1/T_H_17 switches are drawn by extracellular bacteria, viruses, and fungi, and type 2/M2/T_H_2 switches manifest in response to helminth (and other unphagocytosable) infections. Rooted in Eberl's (2016) depiction of immunity's see‐sawing response to stressors, the vigilant and pro‐inflammatory (type 1, type 3) arms of immunity must be followed up with anti‐inflammatory (type 2, type 4) arms for healing and healthy returns to homeostasis. The tissue destructive nature of inflammation means the response is adaptive only when engaged sparingly. A significant challenge facing the modern population, however, is the overabundance of exposures to pro‐inflammatory stressors that regularly dominate or swamp the balance of immunity, stressors drawing potent, ongoing type 1/M1/T_H_1 or, more commonly, type 3/M1/T_H_17 switches, hindering type 2/M2/T_H_2 and type 4/M2/T_H_22 attempts to repair, heal, and protect damaged tissues and organs. Within this scenario, every switch to a pro‐inflammatory state, whether regular or chronic, presents opportunities for latent Mtb reactivation and ongoing cycles of transmission, and while inflammation is expected in response to infections, injuries, surgeries, ovulation, and depression, there are wide numbers of instances in which inflammation arises in the modern population (Burke 2025). In evaluating risks for Mtb reactivation, both generalized and niche‐based, community‐specific pro‐inflammatory stressors should be contemplated.

## 4 How Are Communities Reducing Pro‐Inflammatory Mtb Reactivation Risks?

Community‐specific pro‐inflammatory stressors vary, some common and overlapping with other communities, others more unique. In Indigenous communities, many pro‐inflammatory stressors exist as a result of histories of colonization, with discrimination, marginalization, and trauma creating states of poor nutrition, food insecurity, housing deficiencies, poor sanitation, insufficient access to potable water, high costs of living, and limited access to healthcare (Orr et al. 2024). Because stressors manifesting in this context are typically chronic, there can be downstream impacts on immune function and immune imbalances, particularly if acute inflammation becomes chronic inflammation. In Nunavik communities, drawing on local knowledge and tools like public health education, actions are being taken to reduce known stressors and their impacts. Alcohol and tobacco smoking not only share histories with colonization, they are also potent type 3/M1/T_H_17 pro‐inflammatory stressors which increase risks for latent Mtb reactivation. Courtemanche et al. (2024) note that over the last 30 years “alcohol and drug use in Nunavik have been increasing,” the *Qanuilirpitaa*? (how are we now?) 2017 Nunavik Inuit Health Survey indicating 29% of Nunavimmiut over 16 years of age binge drink (5 or more drinks on one occasion) at least once a week. The survey also emphasizes the critical importance of community supports and *Iqii* (practicing a healthy lifestyle), learning how to manage stressors in different ways (NRBHSS 2024). *Inuit Qaujimajatuqangit* (Inuit traditional knowledge) connects time spent on the land and involvement in community activities with well‐being, encouraging less weekly binge drinking (Courtemanche et al. 2024).

Laneuville (2015) interviewed 108 women (18 to 85 years of age) about various aspects of community life in Nunavik, including the impact of alcohol. The interviews help to place alcohol consumption in context, borne out of difficult circumstances, particularly events beginning in the 1940s with enforced fixed settlement, the kind of life a colonizing and paternalistic government believed Inuit should be living. Explicitly, interviewees explained that alcohol abuse was not just about being forced to become sedentary; that it was also the “many traumatic experiences and rapid changes in Inuit life,” traumas that impacted as “Inuit saw their sled dogs slaughtered and their kids sent off to residential schools, where many of them were molested and sexually abused,” all of that suffering then passed along to the next generations “through violence, alcohol abuse, suicide, and sexual abuse,” such that “now, everyone carries a burden” (Laneuville 2015). In that context, “alcohol became a way to escape reality and to medicate oneself” (Laneuville 2015).

The sled dog slaughter of the 1950s and 1960s left resounding impacts on life in Nunavik.^6^ One 20‐year‐old woman interviewed by Laneuville (2015) came to understand her father's pain and alcohol abuse when she learned more about the community's history, not knowing that when her father was a child, “the RCMP^7^ came and shot their dogs. That was their transportation, dogs were part of the family, the only way to get food.” Understanding the hardships people experienced when their dogs were killed, she said “they just turned to drinking.” Decades later, in 2024, the Government of Canada offered an apology^8^ for the Nunavik dog slaughter, acknowledging the “mass killing of the *qimmiit* (sled dogs) inflicted deep pain and hardship on Inuit families,” the “brutality” leading to “grief and devastation.” Elders described the “horror,” how dogs “were shot, burned in a pile on the ice in front of the owners, … the remains … left there for the winter, forcing the community to be reminded daily of the violence and trauma of losing their dogs.” With a thousand or more killed, the government and police were fully aware the dogs were a “crucial and deeply significant part of Inuit life, allowing hunters to provide for their families and communities, for mobility across the land and sea, and to provide safety and protection” (Government of Canada 2024). The Government of Canada connected the dog killings with an enforced “non‐Inuk way of life,” destroying long‐held traditions and severing “vital connection to the animals, land, and sea.” The Government also acknowledged knowingly allowing the dog slaughter to continue, fully aware the “Inuit way of life, health, and wellbeing depended on the dogs” (Government of Canada 2024).

Like alcohol, a greater reliance on tobacco and cigarette smoking emerged with these traumas and the forced settlement of Inuit. Today, acknowledging “tobacco is harming Nunavik,” the Nunavik Regional Board of Health and Social Services (NRBHSS) maintains a YouTube channel for public health communications and recommendations, allowing for the sharing of personal experiences with cigarette smoking, including Aputik Forrest's heartfelt story (https://www.youtube.com/watch?v=Oq4AJhdI7Y0). Community members are encouraged to quit or “not start” at all, the NRBHSS (2025a, 2025b) emphasizing “traditional Inuit society was smoke‐free and smoking was never a part of Inuit culture.” Bougie and Kohen (2017) estimate 52% Nunavimmiut aged 15 and over smoke cigarettes (compared to 16% of the Canadian population), but the *Qanuilirpitaa*? 2017 Nunavik Inuit Health Survey reports a heavier burden, an estimated 72% of those aged 16 and over smoking cigarettes (NRBHSS 2025b).

If persistent chronic stressors and type 3/M1/T_H_17 inflammation develop into chronic inflammatory diseases, those diseases also become latent Mtb reactivation risks. Large‐scale colonial and capitalist circulations of tobacco and cultures of cigarette smoking have contributed to an epidemic of chronic obstructive pulmonary disease (COPD), the third most common cause of death in the global population (GBD 2023 Causes of Death Collaborators 2025; Herrero‐Cervera et al. 2022). In their study of COPD in a random sample of households in 11 First Nations in British Columbia, Camp et al. (2019) described COPD as “highly prevalent.” Analyzing a 2012 Statistics Canada survey of Indigenous persons 35 and older across Canada, Bird et al. (2017) identified 6.8% of 8117 participants self‐reporting COPD, often in association with daily smoking and additional risks of poor income, aging, and inadequate health care. In Alberta, between 2002 and 2010, First Nations people, Inuit, and Métis had higher COPD prevalence and incidence rates, with First Nations prevalence 2.3 to 2.4 times greater, Inuit 1.86 to 2.10 times greater, and Métis 1.59 to 1.67 times greater than the non‐Indigenous population (Ospina et al. 2015). In COPD, a chronic type 3/M1/T_H_17 immunity switch is engaged, producing ongoing, tissue‐damaging states of inflammation (Lourenço et al. 2021; Rathore and Wang 2016). Like COPD, both type 1 and type 2 diabetes engage chronic type 3/M1/T_H_17 inflammation triggered by insufficient insulin action and tissue‐damaging hyperglycemia (Zhang et al. 2019). In comparison to a type 1/type 2 diabetes prevalence rate of 5% in the general population, rates are estimated at 4.7%, 9.9%, 12.7%, and 17.2% among Inuit, Métis, off‐reserve First Nations people, and on‐reserve First Nations people, respectively (Diabetes Canada 2026; Halseth 2019), demonstrating the variable positioning of diabetes as a niche‐based stressor (much more so among on‐reserve First Nations people than Inuit, for example) influencing latent Mtb reactivation risk across Indigenous communities.^9^

Potential exogenous sources of infection add further risks for pro‐inflammatory type 1/M1/T_H_1 or type 3/M1/T_H_17 immune switches and latent Mtb reactivation. Through subsistence activities like hunting and fishing, Indigenous food systems include a range of traditional (or country) foods, which not only provide key nutrients and help stave off colonization‐related diseases like diabetes, but also foster beneficial connections with the land and nature (Malli et al. 2023). Within Indigenous communities in Canada, the sharing of local knowledge and public health messaging concerning the proper preparation and cooking of country food aim to proactively protect against parasitic infections. Originally an Amazonian protozoan parasite, for example, *Toxoplasma gondii* has become an engrained “Inuit health issue,” an infection that moved into the Arctic ecology via water‐borne oocysts and tissue cysts in infected migratory marine and terrestrial hosts (Reiling and Dixon 2019). Undercooked meat and organs from beluga, seal, and goose, consumption of bivalve mollusk/urchin species, and contaminated natural/municipal drinking water (relative to bottled water) are all potential sources of infection (Ducrocq et al. 2021). Acute toxoplasmosis draws an initial pro‐inflammatory type 1/M1/T_H_1 immune switch before trapping parasites in cysts and settling into latency (with potential disease resurgence if the cysts fail) (Kugler et al. 2016; Latifi and Flegr 2025). Ongoing, chronic toxoplasmosis is connected with type 3/M1/T_H_17 immune switches, neuroinflammation, and mood disorders (Wang, Zhong, et al. 2023). Brucellosis infections are another concern, *Brucella* bacteria known to circulate within Arctic caribou and reindeer populations. In Nunavut, handling and butchering infected caribou, or consuming “raw, frozen, dried or undercooked meat or bone marrow” are risks for human infection (Avatiliqiyikkut Department of Environment 2025). As with toxoplasmosis, an initial pro‐inflammatory type 1/M1/T_H_1 switch is drawn to clear brucellosis infection, with inflammation lingering in convalescent patients even a year after initial onset (Zheng et al. 2019), a “disturbed” or “modulated” T_H_1 response emerging if *Brucella* engages “stealth” strategies and the infection becomes chronic (Ahmed et al. 2016; Skendros and Boura 2013). In either case, the switch away from type 2/M2/T_H_2 immunity and the pro‐inflammatory signaling Mtb receive via vascular connections with granulomas can be read as opportunities for reactivation among well‐adapted bacteria.

Another country food‐related concern, *Trichinella* roundworm infections, occurs with the consumption of infective larvae in raw or undercooked meat. The Government of the Northwest Territories (2025) advises proper cooking and canning of meat, cautioning that freezing meat will not kill the larvae, and that smoking, drying, salting, and microwaving meat are not always effective, listing species such as bear, wolf, fox, wolverine, lynx, walrus, seal, and ground squirrels as potential sources of infection. In Nunavik, undercooked polar bear meat has raised *Trichinella* concerns (Nunavik Department of Public Health 2023). Martinez‐Levasseur et al. (2020) interviewed hunters and Elders from Nunavik concerning safe consumption of locally harvested Atlantic walrus, an important resource helping to buffer against food insecurity. Inuit knowledge has been key to controlling *Trichinella* infections in Nunavik, informing where to hunt for healthy walruses, how to select healthy walruses that are hunted, and how to prepare them for safe consumption. Such knowledge has been combined with community‐based participation in the Nunavik Trichinellosis Prevention Program, also helping to keep the parasite at bay (Larrat et al. 2012). Interestingly, unlike the other country food infections, chronic roundworm infections invoke type 2/M2/T_H_2 immunity (Bruschi et al. 2022) which, in this case, is the anti‐inflammatory switch immunologically aligned with keeping Mtb latent in granulomas.

Indigenous ways of knowing not only help to protect against parasitism in the consumption of harvested food, but also play a critical role in the overall monitoring of animal welfare in conservation, including now “threatened” populations of boreal caribou in the Northwest Territories (Benson and Winbourne 2015). Benson and Winbourne (2015) note that, based on long‐term and careful observations, “hunters and Elders have comprehensive traditional knowledge about past and current caribou populations, movements, health, and habitat,” observing changes in herd demography and features like quantities of back fat, brisket fat, femur marrow fat, and intestinal fat, as well as signs of injuries (including broken bones), evidence of internal and external parasites, and discolored meat, all helping to gauge the wellbeing of individual animals (intended for consumption) and herds (impacted by stressors like mining and hydroelectric projects).

Extending the context of microbial exposures and immunological responses further, a number of bacterial species, some circulating environmentally, others already endogenously established in the microbiome, including *Streptococcus pneumoniae* , *Staphylococcus aureus* , *Klebsiella pneumoniae* , *Mycoplasma pneumoniae* , and group A *Streptococcus pyogenes* , also draw potent pro‐inflammatory type 3/M1/T_H_17 immunity switches if infection is established (Chen et al. 2016; Moffitt et al. 2011; Patel and Kuchroo 2015; Way et al. 2013; Zhang et al. 2023). In community contexts, maintaining balanced controls over these bacteria can be complicated by household overcrowding (leading to greater transmission via respiratory droplets or airborne infections), delayed access to healthcare, and poor sanitation, hygiene, and wound care (very problematic when there is a lack of access to clean water, a critical issue in some Indigenous communities). Kirlew et al.'s (2014) study of CA‐MRSA (community‐acquired methicillin‐resistant *Staphylococcus aureus* ) in on‐reserve First Nation communities in Northwestern Ontario described one community of about 2000 people where only 10% of homes had connections to a water source. If these bacteria establish pathogenic infection and draw type 3/M1/T_H_17 switches, vigilant latent Mtb in granulomas may, once again, become aware of favorable pro‐inflammatory conditions for reactivation.

Deepening the perspective on inflammation‐inducing infections and Mtb reactivation risks, a jointly produced plain language summary by the National Collaborating Centre for Indigenous Health (NCCIH) and the National Collaborating Centre for Infectious Diseases (NCCID) describes “disproportionately high rates” of sexually transmitted and blood‐borne infections (STBBIs) in Indigenous communities in Canada, a situation impacted by systemic and structural limitations in diagnosis, treatment, and prevention plans (NCCIH 2024). STBBIs constitute a pool of chronically circulating infections that can induce type 1/M1/T_H_1 or type 3/M1/T_H_17 pro‐inflammatory switches and also, therefore, create opportunities for latent Mtb reactivation. Of the reportable STBBIs, the rate of chlamydia is “almost 7 times higher among Indigenous adults compared to non‐Indigenous adults,” while Nunavik's 2017 rate of gonorrhea was “28 times higher than Québec's provincial rate,” and Nunavut's 2017 rate of syphilis (234/100000, versus 11.2/100000 nationally) was the highest in Canada. Chlamydia and primary syphilis infections draw pro‐inflammatory type 1/M1/T_H_1 switches (Kaminiów et al. 2024; Redgrove and McLaughlin 2014), while gonorrhea draws a pro‐inflammatory type 3/M1/T_H_17 switch (Scurtu et al. 2022), in all cases pulling immune balance away from the type 2/M2/T_H_2 immunity that helps keep Mtb latent.

Additional potent stressors driving pro‐inflammatory type 3/M1/T_H_17 switches include acute and chronic onsets of anxiety and depression, both amplifying risks for latent Mtb reactivation (Burke 2025). Any sustained adrenalin surges associated with the “predation stress” of anxiety or sustained elevations in cortisol produced under chronic stress can injure tissues and draw pro‐inflammatory responses (Medzhitov 2008). Brinkworth and Shaw (2022) review the ways in which, physiologically, responses to social adversity, including received and perceived discrimination, racism, living with low income, physical harassment, repeated defeat, received cynicism, and post‐traumatic stress disorder, strike connections with strong pro‐inflammatory responses that further damage cells and tissues. Respecting the weight of Indigenous histories of colonialism and abuse, including impacts of residential schools and Indian hospitals and the multigenerational toll on families and communities, mental health and community‐based supports are attempting to mediate these stressors and, in so doing, can also temper the potent, inflammation‐related latent Mtb reactivation risk. Nunavik Elders focus on the importance of going out on the land to manage stress, particularly wishing to share this understanding with youth (NRBHSS 2024).

Collectively, all of these stressors, from alcohol and tobacco, to infections, anxiety, and depression, can engage regular or chronic inflammation and, via a number of different routes and downstream impacts, injure tissues and organs. Whether inducing a type 1/M1/T_H_1 or, more commonly, type 3/M1/T_H_17 immunity switch, inflammation is accompanied by neutrophils, the “most abundant white blood cells in humans” and key “first responders” to pathogens and tissue injuries, drawn by chemokines (chemoattractants) secreted by tissue‐resident macrophages under the influence of epithelial cells and IL‐17 (Herrero‐Cervera et al. 2022; Way et al. 2013). Alongside M1 macrophages, N1 (pro‐inflammatory) neutrophils are a “cornerstone of the innate arm of immunity” involved in both acute (“self‐limiting”) and chronic inflammation, the latter emerging “if the initiating stimulus is not removed or if the resolution program is disturbed” (Herrero‐Cervera et al. 2022; Silva et al. 2023). As inflammation (ideally) resolves once the stressor has passed, anti‐inflammatory M2 macrophages and N2 neutrophils provide post‐inflammatory tissue repair and maintenance (Pothoven et al. 2017). Under the influence of chronic stressors, however, neutrophils can go astray, Herrero‐Cervera et al. (2022) noting the contributions neutrophils make to the pathologies of chronic disease are “significant.”

With a short but variable half‐life that ranges across tissues (about 9 h in the liver, to 18 h in skin), neutrophils emerge continuously from bone marrow, the rate of production matching any increased inflammation‐related demands (Guo et al. 2025; Silva et al. 2023; Zhang et al. 2024). Neutrophils respond to pathogenic infections and tissue damage, undertaking phagocytosis (engulfing and then degrading or eliminating pathogens and cellular debris with reactive oxygen species, antimicrobial peptides and enzymes), degranulation (releasing the contents of their granules, including “an arsenal” of serine proteases, oxidants, and anti‐microbial proteins), and the release of neutrophil extracellular traps (or NETs, “strand‐like webs” of their own DNA and anti‐microbial proteins which entrap bacteria, viruses, and fungi, or anything too large for phagocytosis) which coincide with neutrophil death (NETosis) (Herrero‐Cervera et al. 2022; Silva et al. 2023; Winterbourn et al. 2016; Zhang et al. 2024).

Neutrophils use apoptosis (programmed cell death) to protectively limit their own excessive accumulation (Guo et al. 2025). As macrophages and dendritic cells move towards an anti‐inflammatory switch, IL‐23 and IL‐17 production declines, which then signals for a decline in neutrophil production in bone marrow, a kind of feedback which, according to Guo et al. (2025), “ensures that the number of neutrophils in circulation remains balanced, preventing excessive inflammation and maintaining overall immune health.” Any continued signaling and excessive IL‐17 expression will risk an onslaught of neutrophils arriving on site, a situation in which homeostasis is lost as inflammation becomes pathological and destructive. Otherwise, neutrophils also guide inflammation's resolution with efferocytosis (the clearance of apoptotic neutrophils by macrophages) and the degradation of neutrophil NETs (Herrero‐Cervera et al. 2022).

Like macrophages, neutrophils are also characterized by their “phenotypic plasticity” (Herrero‐Cervera et al. 2022). In chronic inflammation, N1 neutrophils “amplify” inflammation (Herrero‐Cervera et al. 2022), expressing pro‐inflammatory cytokines and chemokines like TNF, IL‐1β, CCL3, CCL5, IL‐6, and IL‐12 (Pothoven et al. 2017), whereas anti‐inflammatory N2 neutrophils reduce inflammation (Zhao et al. 2022). As Herrero‐Cervera et al. (2022) note, disruptions in the pro‐inflammatory to anti‐inflammatory switch draw chronic inflammation and prevent tissue repair and resolution, neutrophils persisting in their pro‐inflammatory activity instead of self‐limiting with neutrophil apoptosis and inflammation resolution (Way et al. 2013). Herein lies the danger of chronic stressors and “persistent” inflammation, neutrophils continuing to release reactive oxygen species and proteolytic enzymes “very damaging to the surrounding tissues,” further sustaining the inflammation (Way et al. 2013).

Along with macrophages, neutrophils release cytokines and chemokines, the signaling molecules that “modulate” other immune cells (Guo et al. 2025). Expressing IL‐8, neutrophils can draw more neutrophils to sites of inflammation, not only “amplifying the inflammatory response,” but also aligned with onsets of inflammatory, infectious, and autoimmune diseases (Guo et al. 2025). Other cell types, including ƴδ T (gamma‐delta T) cells, natural killer cells, natural killer T cells, and some innate lymphoid cells, are also releasing cytokines, including the IL‐17 that underpins neutrophil recruitment, all before T helper (T_H_) cells (adaptive immunity) are engaged, T_H_17 cells differentiating from CD4+ cells, induced by IL‐6 and IL‐1 and activation of the STAT3 pathway (Way et al. 2013). Expression of IL‐17, the signature cytokine of T_H_17 cells, continues under adaptive immunity, T_H_17 cells assuming the “crucial task” of ongoing recruitment of neutrophils to sites of infection or tissue injury (Way et al. 2013). Rathore and Wang (2016) note that if T cell activation is “prolonged or repetitive” they can become “resistant to suppressive cytokines,” making immune switches more difficult. Under persistent, dysregulated inflammation, ongoing IL‐17 expression and neutrophil recruitment become increasingly more “pathogenic” (Way et al. 2013).

Neutrophils are crucially connected to diseases involving chronic inflammation, including type 2 diabetes and chronic obstructive pulmonary disease, both characterized by “expanded subsets” or “accumulations” of neutrophils, along with their damaging output of reactive oxygen species, release of granule contents (including neutrophil elastase and matrix metalloproteinases), and dysregulated NETs formation, creating “persistent tissue damage” (Guo et al. 2025; Herrero‐Cervera et al. 2022). Neutrophils accumulate because of their “prolonged presence,” “delayed apoptosis,” and decreased efferocytosis, causing persistent inflammation, tissue damage, and airway obstruction because of a greater number of active neutrophils releasing twice as much neutrophil elastase and an excess of reactive oxygen species (Guo et al. 2025; Herrero‐Cervera et al. 2022). Serum T_H_17 levels, markers of type 3/M1/T_H_17 inflammation, correlate significantly with the severity of airflow limitation in COPD (Way et al. 2013). Macrophages contribute to the persistence of neutrophils and inflammation, adversely affected by the “excessive oxidative stress” and, in their dysfunction, not able to phagocytose and clear out neutrophils the way they typically would via efferocytosis (Guo et al. 2025). The problem of tissue‐damaging neutrophil accumulations extends to other diseases or disorders involving type 3/M1/T_H_17 inflammation, including obesity, nonalcoholic fatty liver disease, acute respiratory distress syndrome (ARDS), asthma, pulmonary fibrosis, chronic heart failure, diabetes, and atherosclerosis (Guo et al. 2025; He et al. 2020; Herrero‐Cervera et al. 2022; Li et al. 2010; Mills 2023; Wang et al. 2022). Neutrophils have significant impacts in atherosclerosis (chronic large artery inflammation), with NETs contributing to arterial wall plaque formation and neutrophil elastase causing plaque instability, in addition to increased rates of reactive oxygen species production and NETosis (Herrero‐Cervera et al. 2022). Inflammatory conditions can triangulate, the persistent neutrophilic inflammation caused by the hyperglycemia of diabetes also producing atherosclerosis and impaired wound healing, firmly connecting diabetes and cardiovascular disease (Mills 2023).

Between communities, exactly which pro‐inflammatory stressors are present and shaping immunity will likely differ. Pro‐inflammatory stressors are also rarely experienced one at a time, bodies often inducing type 1/M1/T_H_1 and type 3/M1/T_H_17 switches in response to diverse potential stressors, leading to variable pro‐inflammatory switches and states of “dysregulation.” The order of exposure, relative chronicity, and weight of these stressors shape immunity switching, as do individual variation and genetic polymorphisms influencing type 1/M1/T_H_1 and type 3/M1/T_H_17 switch tendencies (including natural variation in degrees of cytokine and chemokine expression and receptor tendencies), but well‐adapted latent Mtb will find opportunities in those switches. The tenets of immune homeostasis suggest pro‐inflammatory stressors are best managed when temporary, with type1/M1/T_H_1 and type 3/M1/T_H_17 inflammation ultimately counterbalanced with the anti‐inflammatory type 2/M2/T_H_2 switches critical for tissue healing. In the context of many modern stressors, however, inflammation is more prevalent and persistent, amplifying risks for Mtb reactivation and transmission. Among Indigenous communities in Canada, because latent Mtb infections are not uncommon, concerns over chronic inflammation and latent Mtb reactivation should be anticipated.

## 5 Why Endemic Mtb (and Other Health Issues) Will Likely Linger Until the Housing Crisis Is Addressed

While Nunavik and Indigenous communities more broadly are working towards reducing the impacts of pro‐inflammatory, immune switching stressors, housing remains a key stressor capable of inducing type 3/M1/T_H_17 inflammation and, because of that, latent Mtb reactivation risk. Nunavik's longstanding housing crisis, defined by acute shortages of homes and poorly engineered homes (most in need of remediation or repair), has made maintaining an adequate housing stock challenging, with culturally‐inappropriate designs and substandard constructions subject to marked “wear and tear” under Arctic extremes (Minich et al. 2011). In Nunavik, the housing shortage “helps to create and perpetuate all major social issues,” including alcohol abuse, and alcohol abuse is connected with violence (Laneuville 2015). Laneuville (2015) notes that in 2006, according to Statistics Canada, “49% of all Inuit in Nunavik inhabited a crowded dwelling (i.e., more than one person per bedroom),” compared with 3% of non‐Indigenous Canadians. Laneuville (2015) notes that while the “high rate of alcohol abuse and violence among Nunavimmiut” emerged out of colonization‐related stressors in the last century, trauma continues to be perpetuated by an ongoing and weighty housing crisis. More so than other pro‐inflammatory stressors, however, mediating the scope and impacts of housing deficiencies falls far beyond the capacities of individual influence and effort (see Lyeo et al. (2024) on the myriad issues for on‐reserve First Nation communities, including limited external funding, mold and moisture issues, and a lack of enforceable building codes).

In their ethnographic study of Nunavut (Canada) and Malmfälten (Sweden), Schmid and Adams (2025) identify persistent problems driving acute housing scarcity in the Arctic, from funding and governance issues, to the logistical challenges involved in building and maintaining homes in the North. There is such a housing crisis in Nunavut that a points‐based wait list has been established for subsidized (public) housing, the unaffordability of housing pushing the roughly 60% of Inuit in subsidized housing in the early 2000s upwards to 86% in 2021 (Schmid and Adams 2025). Families with children are awarded more points and placed higher on the wait list than families without children, but Nunavut's high fertility means the population is growing, further aggravating the housing crisis. Inadequate federal government funding is central to housing scarcity and homes falling into disrepair, the issue first emerging in the 1940s as Inuit were forced into settlements without access to proper housing (Schmid and Adams 2025). In addition to insufficient fiduciary support, the logistics of growing and maintaining a housing stock in the Arctic is riddled with challenges, from shortages of builders, to limited, annual arrivals of cargo ships responsible for importing all building materials (while also needing to prioritize critical food and fuel shipments) (Schmid and Adams 2025). The building and repairs season in the High North is typically restricted to June through September, dealing with everything from burst pipes and indoor flooding (frequent in the month of February), to broken glass windows ‘temporarily’ replaced with “plastic garbage bags taped across window frames” in temperatures of −40°C or more, and homes built on long steel poles becoming destabilized by climate change‐related thawing of the permafrost (Schmid and Adams 2025). The pace of construction is often limited by difficulties accessing building materials and diesel (to run machines and trucks). For all these reasons, carefully heard and documented by Schmid and Adams (2025), “there are simply not enough buildings to house Nunavut's residents,” with overcrowding and wear and tear a constant grind.

Surveys of Inuit homes reveal abundant physical and mental health issues connected with high levels of crowding, including feelings of unhappiness, anger, and irritability, one survey reporting about 54% of Inuit children growing up in crowded conditions (Minich et al. 2011). This situation contributes to risks of domestic violence, another factor also given points on Nunavut's housing wait list (Schmid and Adams 2025). Overcrowding and poor indoor air quality contribute to a high prevalence of poor respiratory health, from wheezing, shortness of breath, chronic obstructive pulmonary disease, and asthma, to tuberculosis, respiratory syncytial virus (RSV), pneumonia, chronic bronchitis, other upper and lower respiratory tract infections, and lung cancer (Anwar et al. 2021; Hyslop et al. 2025; Kilabuk et al. 2019; Kovesi et al. 2022; Orr et al. 2024; Waapihk Research 2024). A study of two Saskatchewan First Nations identified associations between chronic bronchitis and the odor or musty smell of mildew/mold and environmental tobacco smoke in the house (Pahwa et al. 2017). According to Kovesi (2012), children living in Nunavut's Baffin (Qikiqtani) Region experience “the highest known rates of RSV bronchiolitis requiring hospitalization.” Woo Kinshella et al. (2025) undertook a 10‐year retrospective cohort study of infants (under 1 year) from Nunavut hospitalized for acute respiratory tract infection (ARI) between 2010 and 2020, noting 1189 admissions and an incidence rate of 133.9 ARI‐related hospitalizations per 1000 infants per year. Of the ARI admissions, respiratory syncytial virus (RSV) was described as a “leading pathogen,” yielding a yearly RSV incidence rate of 37.8/1000 infants, in comparison to 15.9/1000 for the global population and 10.2/1000 for the province of Ontario (Woo Kinshella et al. 2025). Infants admitted with RSV (versus other respiratory tract infections) were at higher odds for intensive care, oxygen therapy, CPAP/BiPAP respiratory support, and hospitalizations lasting more than 1 week. Reviewing the literature, Woo Kinshella et al. (2025) contemplate “underlying reasons” for the high rate of infant ARI‐related hospitalizations, including social determinants such as food insecurity, household overcrowding, poor indoor air quality, along with potential genetic determinants and difficulties accessing medical care in remote communities.

Overcrowded and poorly ventilated homes not only facilitate infection transmission, they also draw chronic, everyday pro‐inflammatory type 3/M1/T_H_17 immune switches (to the detriment of type 4/M2/T_H_22 respiratory mucosal barrier protections), increasing not only Mtb reactivation risk but a wealth of health concerns. Inflammation risks stemming from housing are reviewed by Brinkworth and Shaw (2022), connecting wealth inequality, poor living conditions, and the increased risk of sepsis, an extreme immune response to infection with widespread, tissue‐damaging inflammation and mortality risk. Brinkworth and Shaw (2022) describe how race, poverty, and substandard housing intersect in older, poorly ventilated homes with high concentrations of allergens, endotoxins, volatile organic compounds, pests, and lead, adding further to immune challenges already brought on by reduced access to preventive care and chronic social adversity. Noting a common thread across Anglocolonial states, Brinkworth and Shaw (2022) describe how crowding, inadequate ventilation, poor cleaning facilities, and the absence of running water strongly associate with increased sepsis risk among Indigenous peoples, often living in homes in communities under governmental fiduciary responsibility.

With a focus on the autonomic nervous system and the hypothalamic–pituitary–adrenal (HPA) axis, Brinkworth and Shaw (2022) outline the biology connecting the chronic stress of social adversity with sepsis, noting that under the “flight or fight response,” there can be “stress‐induced white blood cell programming” which notably “shifts towards continuous proinflammatory cytokine expression” with downstream effects creating further dysfunction. In this context, Brinkworth and Shaw (2022) describe how the acute pro‐inflammatory triggering of innate immunity under stress can have benefits “when a fleeing host might be injured and have an acute need to stave off incoming microorganisms,” but how, under chronic, unrelenting stress, there is “continuous and damaging immune activation” manifesting in chronic inflammation. In their argument, anxiety linked to social adversity and housing insufficiencies is a key stressor inducing dangerous immune‐compromising states of chronic inflammation that can lead to sepsis.

In addition to stress, Brinkworth and Shaw (2022) describe housing's intersections with other inducers of inflammation, including endotoxins. Endotoxins are immune‐stimulating lipopolysaccharide residues from the outer membranes of dead Gram‐negative bacteria that have “strong immunostimulatory” properties when inhaled, driving alveolar macrophages to encourage type 3/M1/T_H_17 pro‐inflammatory switches in the respiratory tract (Glader et al. 2010; Sakaguchi et al. 2016; Zhao et al. 2024). Endotoxins are not live pathogens, only bits of leftover pathogenic signatures, but by inducing inflammation, they may chronically or regularly draw the balance of immunity away from latent Mtb reactivation‐restraining type 2/M2/T_H_2 immunity. While endotoxins are generally common in homes and other indoor environments, unduly high concentrations can accumulate in dust and air (Zhao et al. 2024). Overcrowding, indoor tobacco smoking, and pets may cause greater endotoxin accumulations that can be offset with good quality ventilation from well‐functioning exhaust fans or open windows (if environmentally feasible) (Zhao et al. 2024). In addition to endotoxins, chronic fungal exposures in homes also draw type 3/M1/T_H_17 immune switches due to immune‐stimulating β‐glucan, chitin, and mannan in the cell walls of mold, mushrooms, and yeast, respectively (Levitz 2009; Yoshida et al. 2012). Cigarette smoke contributes further to inflammation in homes, interfering with the work of macrophages and neutrophils (Herrero‐Cervera et al. 2022). Cigarette smoke is characterized as a “strong T_H_17 adjuvant,” signaling its ability to induce inflammation (Strzelak et al. 2018; Way et al. 2013). While T_H_17 cells and IL‐17 cytokine are not inherently pathological, the chronic presence of cigarette smoke drawing their chronic expression is pathological such that, so long as the smoke is present, so is the risk for type 3/M1/T_H_17 inflammation.

As a case in point, T_H_17 cells and IL‐17 are “protective” against fungal infections, including blastomycosis, coccidioidomycosis, and histoplasmosis (Way et al. 2013). Experimental mice low in T_H_17‐related cytokines (including IL‐17) were more vulnerable to fungal infections ( *Candida albicans* , *Aspergillus fumigatus* , *Blastomyces dermatitidis*) because of compromised neutrophil recruitment to infection sites (Rathore and Wang 2016). While T_H_17 responses and cytokines offer protective functions, under any “defect” or dysregulation, they can become “pathogenic” (Way et al. 2013). “Excessive” IL‐17 secretion, pathologically high neutrophil recruitment and activation, and the dysfunction caused by “neutrophilia” sacrifice the otherwise protective actions of neutrophils managing fungal exposures and preventing infections, leading to fungal translocations across mucosal barriers and potential invasive infections (Mills 2023). Under chronic exposure to fungi, the ongoing switch for T_H_17 cells and IL‐17 will drive ongoing neutrophil recruitment in the lungs, increasing their tissue‐damaging release of protease enzymes (neutrophil elastases and matrix metalloproteinases) (Way et al. 2013). Ongoing inflammation may create other disease risks, such as respiratory syncytial virus (RSV) infections in lungs because of the dysfunction that arises out of excess neutrophil accumulations (Way et al. 2013). As a result, chronic everyday exposures to fungi, endotoxins, and tobacco smoke keep type 3/M1/T_H_17 inflammation engaged, risking not only Mtb reactivation, but also a greater vulnerability to infections.

First Nations reserves located in damp, low‐lying, flood‐prone areas of coastal British Columbia can be affected by household mold, but so can homes in drier Arctic Inuit villages if chronically damp conditions persist within homes (Kovesi 2012). Estimates from the 2007–2008 *Qanuippitali?* (How about us, how are we?) Inuit Health Survey of 1901 households revealed 25% of children growing up in homes with mold (Minich et al. 2011). In a Manitoba First Nations research partnership, Larcombe et al. (2011) surveyed housing quality in two communities concerned about tuberculosis and other infectious diseases, one experiencing ongoing endemic tuberculosis transmission with epidemic potential. Fungal problems were documented, with mold readily observed in 44% of homes in one community, the captions on two figures reading “mould growth around a non‐operational ceiling fan,” and “fungi growing in the crawlspace of a bungalow in a Dené house in early June,” the image capturing a significant bloom of mushrooms with lanky, drawn‐out stems (Larcombe et al. 2011). More broadly, surveys of Indigenous homes in Canada reveal a pervasive “mold crisis” on reserves and in villages due to inadequate insulation and thermal bridging, structural damage to building envelopes, and showering, cooking, and human respiration in overcrowded homes with insufficient ventilation, all producing high levels of fungus‐inducing humidity and dampness inside (Optis et al. 2012).

Reviewing 2006 Statistics Canada data, Optis et al. (2012) note that 44% and 26% of on‐reserve First Nation homes were in need of repair and overcrowded, respectively, in comparison to 7% and 3% of non‐Indigenous homes in Canada. Optis et al. (2012) argue the pervasive mold problem is a byproduct not only of poor choices in construction materials, but also the “political and economic environment,” originally staked in the 1860s with the onset of forced removals from traditional territories. In their review, Optis et al. (2012) describe problems created by the replacement of traditional homes “built with local materials and techniques” with inadequate and ill‐suited “European‐style colonial dwellings” built with “inappropriate materials” like poorly dried (“green”) wood and untreated gypsum wallboard. Dillon Consulting (2025) was retained by the Nunavut Housing Corporation in 2017 to assess over 500 housing units across Nunavut, the review producing “a ‘tool box’ of design strategies for the building envelop and mechanical ventilation systems” that could be applied in mold remediation efforts and to improve the quality of future new builds. Larcombe et al. (2020) mobilized an approach drawing together community members of the Northlands Denesuline and Sayisi Dene First Nations in Manitoba with University of Manitoba Architecture students in a culturally‐ and environmentally‐informed collaboration involving reciprocal field experiences in the development of innovative housing designs (see Larcombe and Coar 2018). Optis et al. (2012) suggest mold remediation in band‐owned housing^10^ is complex, considered a Band Council^11^ responsibility which, then, due to limiting funding, hinders remediation efforts. As Larcombe et al. (2020) argue, “while it is relatively easy to identify what is wrong” with housing in Indigenous communities, acting upon those problems is often hampered by “politics, economics and historical management processes at all levels,” from local communities to the federal government. Hyslop et al. (2025) affirm the federal government's “weight of responsibility for housing designs and conditions on‐reserve,” a situation characterized by “years of insufficient funding, low‐quality building materials, and lack of First Nations' control.” In moving towards reconciliation, Hyslop et al. (2025) argue, reducing or eliminating housing‐related health risks will require “sufficient” and “sustained” funding and Indigenous controls.

In research, Larcombe et al. (2011) suggest, “it is difficult to demonstrate a direct causal relationship between select illnesses, such as tuberculosis, and inadequate housing.” Via diverse stressor‐driven type 1/M1/T_H_1 and type 3/M1/T_H_17 immune switches, including chronic everyday exposures to fungi and endotoxin in homes, this paper argues real and tangible immunological connections between inflammation‐inducing household exposures and latent Mtb reactivation risk, such that improving housing is a critical lynchpin upon which the “End TB” tuberculosis eradication efforts rest. While the effects of poor ventilation and overcrowding on tuberculosis infection transmission within households have long been known, chronic endotoxin and fungi exposures add to the collective of pro‐inflammatory stressors which drive potent and chronic pro‐inflammatory type 3/M1/T_H_17 immunity switches and create real immunological opportunities for latent Mtb reactivation. In this way, housing‐induced type 3/M1/T_H_17 pro‐inflammatory switches are not mere background factors, or even “immunosuppressing” in tuberculosis risk, but actual immunological instigators of both latent reactivation (since latency requires regular type 2/M2/T_H_2 immune balances) and increased vulnerability to primary infection (which requires a type 1/M1/T_H_1 response) among those exposed to reactivated cases in homes and communities. By attending to inadequate housing and reducing burdens of exposures to fungi and endotoxins, the weight of the stressors inducing type 3/M1/T_H_17 switches would be lightened.

## 6 Furthering Chronic Type 3/M1/T_H_17 Pro‐Inflammatory Risks: Compromised Mucosal Barriers, Severe Asthma, and Translocating and Invasive (Bacterial, Viral, Fungal) Infections

While chronic type 3/M1/T_H_17 inflammation risks latent Mtb reactivation, other aspects of immune function, including mucosal barrier protections, can also be impacted. The mucosa lining the respiratory (from nose to lungs), digestive (from mouth to lower gastrointestinal tract), and urogenital tracts and the eyes (the conjunctiva) connect with Eberl's (2016) fourth protective arm of immunity, an arm which “aims to block” infections and toxins from reaching sensitive tissues, preventing harmful states of inflammation in sensitive organs like the lungs, brain, and eyes. Mucosal barriers are multi‐layered systems under continuous exposure to the “outside environment,” needing to maintain “local microenvironment homeostasis and systemic health,” while handling the microbiome and diverse “exogenous antigens” breathed into the respiratory tract, taken into the digestive tract, or passed between bodies (with sexual intercourse) (Silva et al. 2023; Song et al. 2023). Mucosal tissues exist in a changing balance, maintaining their integrity while regulating local inflammatory responses to stressors, including infection and injury (Silva et al. 2023).

Mucosal barriers must be judicious, working with innate and adaptive immunity to protect against infections, while providing a “habitat” (complete with nutrients and adhesion sites) and support for the symbiotic and advantageous microbiome, always attempting to avoid unnecessary inflammation to harmless antigens (Hall et al. 2017; Kang et al. 2022; Paone and Cani 2020; Schnell et al. 2023; Song et al. 2023). In the shifting balance of immunity, however, ongoing, regular, or even chronic pro‐inflammatory stressors, including the type 3/M1/T_H_17 switches drawn by stressors like fungi, endotoxins, alcohol, and depression, may compromise healthy mucosal barrier function. If barrier dynamics are compromised, risks like severe asthma and translocated (breaching the protective mucosal barrier) pathogenic and potentially invasive infections can be considered, alongside reactivations of latent Mtb infections, as another dimension of harm created by chronic type 3/M1/T_H_17 inflammation.

In “immunity by equilibrium,” Eberl (2016) acknowledges the innate and adaptive protections at play with the type 4 arm of mucosal immunity. The lumen‐facing side of the mucosal barrier makes first contact with antigens and microbiota, the outer layer composed of a thick mucin gel, under which resides an epithelial cell layer with exclusionary tight junctions, and below that a deeper lamina propria layer (home to cells involved in innate and adaptive immunity), each layer of defense meant to prevent pathogenic incursions past the mucosa and into the bloodstream and tissues (Chen and Yao 2023; Leceta et al. 2022). The lamina propria is made up of loose connective tissue, lymphoid tissue, blood and lymphatic vessels, and nerves, and provides structural support, attached to the epithelial layer and grounded to the muscle layer below, the deepest layer, providing the “motor function” that keeps “the mucosa in motion,” stretching and contracting with the organs (Song et al. 2024). The mucosal barrier is “dynamic and regulated,” the layers working together, selectively controlling movement of lumen contents into the lamina propria (Hall et al. 2017), while also protecting against mechanical, chemical, and biological agents and preserving homeostasis (Paone and Cani 2020; Song et al. 2023). Mucosal barriers work with the immune system, employing specialized microfold cells (a subtype of epithelial cell) to sample antigens, microbiota, and particles encountered with breathing, eating, and contact, drawing them in for evaluation and immunosurveillance in the lamina propria (Al‐Talib et al. 2024; Song et al. 2024).

At steady state, mucosal barriers are anti‐inflammatory and can be characterized by a type 4/M2/T_H_22 composite, acknowledging the type 4 arm outlined by Eberl (2016), the regular tissue‐resident presence of anti‐inflammatory M2 polarized macrophages in the lamina propria (Kagoshima et al. 2025; Smith et al. 2011) (versus the M1 “infiltration macrophages” that arrive if microbiota, toxins, or antigens trigger inflammation; Meng et al. 2024; Smith et al. 2011), and the T_H_22 CD4+ T cells also in the lamina propria (T_H_22 cells are specifically characterized as mucosal T helper cells, Chen and Yao 2023). Xu et al. (2014) describe T_H_22 cells in mucosa and the IL‐22 they secrete as “crucial” for host protection. Mucosal barriers are best positioned in an anti‐inflammatory steady state, with careful use of inflammation, helping to preserve the microbiome and avoid unnecessary tissue damage. In Eberl's (2016) four‐way crisscrossed see‐saw of immunity, anti‐inflammatory and reparative type 4/M2/T_H_22 immunity counterbalances against anti‐inflammatory and reparative type 2/M2/T_H_2 immunity, and both are offset from the counterbalancing pro‐inflammatory arms of type 1/M1/T_H_1 and type 3/M1/T_H_17 immunity. Because of the principle that whenever one arm of immunity is engaged, the others are repressed, chronic pro‐inflammatory switches can problematically hinder periodic switches to type 4/M2/T_H_22 immunity needed for mucosal barrier repair and maintenance. While a range of cells can produce IL‐22, including T_H_1 and T_H_17 cells, ƴδ T cells, natural killer T cells, and innate lymphoid cells, T_H_22 cells are most prolific in IL‐22 secretion (Klotskova et al. 2024). IL‐22 stimulates epithelial cell proliferation and mucin production, the cytokine playing a key role “maintaining the mucus layer,” supporting and housekeeping the mucosa with epithelial layer repairs and activating DNA damage response pathways to protect against genotoxic stress (Klotskova et al. 2024).

The outermost‐facing mucus gel layer is composed of water, electrolytes, lipids, and proteins (mucin being the key contributor), providing a thick and viscous “coat” that covers the epithelial cells, “protecting them from contact with external and toxic substances, digestive enzymes, and bacteria,” while also serving as a “surface cleaner” for the removal of “debris and bacteria” by “binding, collecting and flushing them away” (Paone and Cani 2020). Goblet cells are specialized epithelial cells that produce the mucin for the “gel‐like mesh structure,” the secretion continuous and the barrier under ongoing “homeostatic maintenance,” refreshed with mucin (Chen and Yao 2023; Cornick et al. 2015; Llorente and Karin 2025; Meng et al. 2024; Song et al. 2023). In addition to protecting the epithelial cells from pathogens and “noxious substances,” the mucus gel nourishes the microbiome (Cornick et al. 2015; Paone and Cani 2020; Song et al. 2023).

The mucus gel is populated with antimicrobial peptides and immunoglobulins like secreted IgA (sIgA), the “most abundantly produced antibody at mucosal surfaces,” both able to bind antigens from pathogens and microbiome, food, and environmental toxins to the mucosal surface, preventing penetration and protecting the epithelium, cleared away by phagocytic cells like neutrophils and dendritic cells, all without engaging inflammation (El Ansari et al. 2025; Mantis et al. 2011; Meng et al. 2024; Song et al. 2023; Winstead 2014). Some symbiont bacteria possess adhesins that bind to mucin, benefitting them for the stationary grounding, while also benefiting the host by reducing their access to the epithelium (Cornick et al. 2015; Smith et al. 2023). Members of the microbiome that reside in the mucus gel layer also “occupy the empty niches,” restricting possibilities for pathogenic colonizations (Cornick et al. 2015). If the mucus layer is compromised, if there is any “impairment” or “loss” to the gel matrix, the underlying epithelial cells may be exposed to microbiome species and pathogenic microbes, both with disease‐causing potential (the pathogenic microbes by nature, and the microbiota because they are no longer being effectively governed by innate immunity) (Song et al. 2023).

From microbes and microbial products to toxins and cytokines, Cornick et al. (2015) note the gel mucus layer can be manipulated and compromised. Some pathogens “subvert” and “penetrate” the barrier, decomposing mucin with the release of proteases (enzymes that cleave glycoproteins like mucin) (Cornick et al. 2015; Song et al. 2023). Microbes with flagella may use their appendages as mucin adhesins or to “burrow through the mucus layer” (Cornick et al. 2015), the mucosa's epithelial cells equipped to respond with Lypd8, a protein which binds to flagella, inhibiting bacterial movement and preventing penetration (Song et al. 2023). Other pathogens manipulate mucin secretion from the goblet cells, increasing or inhibiting production. The lipopolysaccharide component of Gram‐negative bacterial cell membranes and the cholera bacterium's toxin encourage mucin secretion to the degree that goblet cells become depleted of mucin, exposing the epithelial cells to infection (Cornick et al. 2015). With mucin secretion, the protective tight junctions between epithelial cells also loosen, creating another vulnerability to infection (Cornick et al. 2015). In contrast, *Clostridium difficile* inhibits mucin secretion in order to access the epithelial cells (Cornick et al. 2015). While some pathogens aim to penetrate the barrier, others may just do so opportunistically via microabrasions (repaired by type 4/M2/T_H_22 switches), or getting caught up and moved by dendritic cells whose dendrites extend into the lumen to sample pathogens and antigens (Bogunovic et al. 2009; Song et al. 2024). Janoff et al. (2014) describe virulence factors possessed by mucosal pathogens, including *Streptococcus pneumoniae* , *Neisseria* species, and *Haemophilus influenzae* , secreting IgA‐cleaving proteases that enhance invasive infection potential, to which hosts may respond with protease‐inhibiting IgG antibodies.

Below the mucin is the barrier's epithelium, often “just a single layer of epithelial cells” separating environment from interior, a selective barrier that protects from “pathogens and other potentially harmful factors,” while also allowing for ingress and egress of nutrients and gases (Chen and Yao 2023; Song et al. 2024). Beyond mucin secretion, epithelial goblet cells are also key immune regulators, secreting cytokines (including the IL‐17 that prompts macrophages to release neutrophil‐recruiting chemokines), chemokines, and antimicrobial peptides, supporting returns to homeostasis (Llorente and Karin 2025). In the intestinal mucosa, epithelial cells possess “an enormous self‐renewing capacity” and a “high cell turnover rate,” emerging out of stem cells in epithelial crypts, able to differentiate into variable types of epithelial cells (some ciliated to help move mucus) and then “shed into the intestinal lumen after 4‐5 days,” the regular “sloughing off of intestinal mucosal cells” helping to maintain the epithelium's integrity and strengths against pathogen invasion (Ruder and Becker 2020; Song et al. 2024; Winter et al. 2014). Because of these multiple roles, epithelial cells are more than just “physical barriers,” assisting in “immune surveillance” and engaging with innate and adaptive immunity (Hall et al. 2017; Song et al. 2024).

Macrophages offer more innate immune protections in the mucosa, resident in the subepithelial lamina propria which is home to “the largest number of macrophages in the body” (Ruder and Becker 2020). Macrophages are prepared to phagocytose anything which might translocate the epithelial layer, while attempting to maintain a restrained “anti‐inflammatory profile” to protect the microbiome (Ruder and Becker 2020). Macrophages phagocytose pathogens, apoptotic cells, and cellular debris, while also producing antimicrobial peptides and secreting factors that support the microbiome (Meng et al. 2024). Maintaining a supportive but vigilant anti‐inflammatory state and “a stable and balanced microbial community,” macrophages help to preserve “gut barrier integrity” and “immune homeostasis” (Meng et al. 2024). When exposed to antigens, macrophages and dendritic cells “decompose the antigen into fragments” and, downstream, activate IgA secretion by B cells which are also resident in the lamina propria (Song et al. 2023). Macrophages secrete growth factors promoting rapid proliferation and differentiation of epithelial cells, aiding in wound healing and mucosal barrier integrity (Meng et al. 2024). In addition, macrophages secrete cytokines and chemokines and present antigens to T cells and, in so doing, can initiate and regulate the pro‐ or anti‐inflammatory state and T_H_ cell balance of adaptive immunity (Meng et al. 2024; Ruder and Becker 2020).

Under steady state conditions in the digestive tract, resident macrophages in the lamina propria secrete IL‐10, a potent anti‐inflammatory cytokine which helps to suppress “excessive inflammatory activity” and supports a “tolerant environment in the gut,” while also maintaining the protective tight junctions between epithelial cells (Meng et al. 2024). Macrophages secreting IL‐10 and TGF‐β (also inflammation controlling) collaborate with regulatory T cells (Tregs) to suppress inflammation and produce tissue repairing proteins (Proto et al. 2018). Another subset of macrophages in the lamina propria secretes IL‐1β, which supports development of T_H_17 cells (Ruder and Becker 2020). If the digestive tract mucosa is damaged by “invading pathogens” or toxins, carefully moderated inflammation triggers recruitment of M1 (pro‐inflammatory) infiltrating macrophages to the lamina propria which, in the aftermath, as the inflammation resolves, repolarize to an M2 (anti‐inflammatory) state in support of regeneration and repair of epithelial cells and mucosal wounds (Meng et al. 2024; Ruder and Becker 2020). According to Meng et al. (2024), chronic inflammation and a prolonged persistence of M1 macrophages can cause “barrier depletion,” compromising the epithelium's tight junctions and, via the permeability, allow pathogens and toxins to translocate the epithelium and access the bloodstream (notably, on the flip side, sustained overactivation of M2 macrophages can cause problems of fibrosis and poor pathogen clearance). Otherwise, well modulated M2 macrophages that suppress inflammation are associated with the type 4/M2/T_H_22 switches needed for barrier repair and maintenance.

Along with macrophages, neutrophils are also “sentinel cells” contributing to innate immune defenses in the mucosa (Silva et al. 2023). At steady state, N2 (anti‐inflammatory) neutrophils assist in the maintenance of tissue homeostasis and, in the respiratory tract, N2 neutrophils are found “adhering to the endothelium of capillaries and postcapillary venules in the lung” (Silva et al. 2023). N1 (pro‐inflammatory) neutrophil recruitment is typically suppressed by the microbiome, preventing neutrophil accumulations in steady state conditions (Silva et al. 2023). The IL‐17 expressed by epithelial cells (and T_H_17 cells if adaptive immunity is activated) recruits neutrophils which originate in bone marrow and are delivered by blood vessels to the lamina propria, considered the “first line of defense” in infection (Hall et al. 2017), ideally not too many (neutrophilia) nor too few (neutropenia) but a “balanced neutrophil response” (Silva et al. 2023). While other immune cells in the lamina propria are engaged in sampling lumen contents and immunosurveillance, neutrophils actually cross the epithelial layer, a transepithelial migration from lamina propria to lumen, to become directly involved and proactive in phagocytosing pathogens and cellular debris and other matter (Denning and Parkos 2013). The only problem with this response is if it becomes unduly regular or chronic since the migration of neutrophils does cause epithelial injury, disrupting the barrier to the extent that more neutrophils have to be recruited because of “enhanced leakage of luminal contents into the mucosa” (Denning and Parkos 2013). Chin and Parkos (2007) note that ongoing neutrophil transepithelial migration is “a hallmark of many inflammatory conditions,” and “correlates directly with clinical disease activity and epithelial injury.” Ultimately, “excessive neutrophil infiltration” is a problem, with antimicrobial and cytotoxic agents expressed by neutrophils, causing “significant epithelial injury” as degranulating neutrophils release epithelial barrier protein‐degrading neutrophil elastase, compromising the epithelial tight junctions and causing “enhanced permeability” (Chin and Parkos 2007; Denning and Parkos 2013). Denning and Parkos (2013) suggest that if neutrophils continued to be recruited and activated “unabated,” mucosa would come to experience “severe and potentially life‐threatening damage.”

Rapid recruitment of N1 (pro‐inflammatory) neutrophils followed by efficient resolution and healing with N2 (anti‐inflammatory) neutrophils is critical, both polarizations expressing “a broad range” of cytokines and chemokines (Hall et al. 2017). Once their work is complete, once the stressor has (ideally) passed, neutrophils initiate the timely resolution of inflammation, releasing agents like lipoxins, resolvins, and protectins to stop the recruitment of neutrophils (Denning and Parkos 2013), and undergoing apoptotic cell death and releasing “‘find me’ signals” to draw in macrophages for efferocytosis (Silva et al. 2023). Efferocytosis, in turn, triggers macrophages to repolarize from an M1 (pro‐inflammatory) to M2 (anti‐inflammatory) state, guiding innate immunity's transition from inflammation to healing and then steady state conditions (Silva et al. 2023). Neutrophils phagocytosing other apoptotic neutrophils (“neutrophil cannibalism”) is also observed in the resolution of inflammation (Ramos and Oehler 2024).

Cytokines, secreted by a range of cells, guide these barrier dynamics. Natural killer T cells (NKT), for example, are innate immune cells known for “regulating the transcriptional and cellular landscapes of the intestinal epithelium,” secreting anti‐inflammatory IL‐4 at steady state, regulating memory CD8 T cells, IgE production by B cells, and chemokine secretion by dendritic cells (Lebrusant‐Fernandez et al. 2024). When exposed to antigens, however, NKT cells express IFN‐ƴ, a pro‐inflammatory cytokine (Lebrusant‐Fernandez et al. 2024). In addition to natural killer T cells, natural killer cells, macrophages, neutrophils, innate lymphoid cells, and other innate immune cells, as well as epithelial cells, express a range of pro‐ and anti‐inflammatory cytokines, including IL‐17 and IL‐22, two critical mucosal cytokines contributing to mucosal defenses and homeostasis, both able to recruit neutrophils and drive antimicrobial peptide production, even though these cytokines emerge from different lineages (Xu et al. 2014). IL‐17 is the more common or potent neutrophil recruiter, while IL‐22 is notable for increasing the mucosa's immune defenses, enhancing tissue regeneration and protecting against tissue damage (Valeri and Raffatellu 2016). While IL‐17 typically drives N1 neutrophil recruitment with the onset of inflammation, N2 neutrophils secrete the IL‐22 which prompts the switch to anti‐inflammatory healing, resolving the inflammation (Xie et al. 2023; Zindl et al. 2013). Valeri and Raffatellu (2016) acknowledge the balance, IL‐17 inflammation‐inducing, and IL‐22 “largely protective and regenerative.” Zindl et al. (2013) also describe the synergy, IL‐17's work in inflammation counter‐balanced by IL‐22's “protective effects on the epithelium,” one cytokine causing tissue damage responding to stressors, the other then providing healing and maintenance. Because both cytokines are secreted by a range of innate cells in the mucosa, this ensures an efficient initial response, even before adaptive immunity and CD4+ T helper cells are engaged (and perhaps even preventing their engagement altogether, beneficial since adaptive immunity's inflammation is best avoided) (Basu et al. 2012).

In terms of CD4+ T helper cells in the lamina propria, IL‐17 and IL‐22 are the signature cytokines of T_H_17 (contributing to barrier protections and specialized for extracellular pathogens) and T_H_22 (contributing to barrier maintenance and repair) cells, respectively. Also in the lamina propria and contributing to “immune vigilance” at mucosal barriers are T_H_1 (intracellular pathogens), T_H_2 (helminths and tissue repair), T_FH_ (helper cells for B cell follicles) cells, and regulatory T cells (Treg) (possessing anti‐inflammatory effects), all existing in a shifting balance, engaging type 1/M1/T_H_1, type 2/M2/T_H_2, or type 3/M1/T_H_17 immunity as needed, depending on stressors and circumstances (Lebrusant‐Fernandez et al. 2024; Leceta et al. 2022).

Mucosal immunity settles into an anti‐inflammatory type 4/M2/T_H_22 immunity at steady state, but with an “abundant” and “homeostatic” T_H_17 cell population supporting barrier function and mucosal immunity (Schnell et al. 2023; Song et al. 2024). The IL‐17 expressed by T_H_17 cells also stimulates the secretion of proteins involved in maintaining the epithelium's tight junctions, and regulates sIgA antibody production by B cells, deficiencies in T_H_17 cells adversely impacting antibody presence (Cao et al. 2012; Schnell et al. 2023; Valeri and Raffatellu 2016; Wu et al. 2016; Xu et al. 2014). In the urogenital tract, IL‐17 protects against *Chlamydia* infection, elevating nitric oxide production by macrophages, while in the upper respiratory tract IL‐17 recruits phagocytic neutrophils to prevent nasopharyngeal colonization by *Streptococcus pneumoniae* (Mills 2023). T_H_17 cells can also prompt IL‐26 expression, a cytokine that causes pores to form in infected cells, holes in their cell membranes through which cell‐destroying molecules gain access (Mills 2023). When inflammation is triggered by mucosal stressors, there is an expansion of T_H_17 cells in the lamina propria, and an increase in IL‐17 secretion connected with a suite of pro‐inflammatory actions (Kempski et al. 2017; Valeri and Raffatellu 2016).

In comparison, IL‐22 is noted for its role in “immunosurveillance” and “preserving mucosal immunity” (Chen and Yao 2023; Valeri and Raffatellu 2016), Klotskova et al. (2024) describing the cytokine's ability to induce epithelial cells to secrete “just the right amount” of antimicrobial peptides to “ward off” microbes at mucosal surfaces without disturbing the microbiome. Described as “the cytokine of epithelium protection” and “barrier integrity,” IL‐22 is acknowledged for its “tissue‐protective properties,” supporting epithelial wound healing via epithelial cell proliferation and increased goblet cell mucin production in the “rapid alleviation” of mucosal inflammation (Broquet et al. 2017; Cornick et al. 2015; Denning and Parkos 2013; Valeri and Raffatellu 2016; Xu et al. 2014). Acting to repair lung mucosa after influenza, IL‐22 reduces susceptibility to subsequent secondary bacterial infection (Way et al. 2013). Valeri and Raffatellu (2016) and Klotskova et al. (2024) emphasize the importance of IL‐22's impact on epithelial cell proliferation in the intestine, “maintaining and restoring epithelial barrier function,” a critical contribution “since the rapid renewal of the epithelium is the foundation of the symbiotic relationship between gut microbiota and the host,” otherwise intestinal microbes could “overwhelm the host and its immune system.” While IL‐22 can assist in recruiting neutrophils, more significantly Broquet et al. (2017) describe the cytokine's role in “moderating” neutrophil accumulations and protecting the epithelium, their study demonstrating that neutralizing IL‐22 during pneumonia infection produced greater neutrophil recruitment and lung lesions. Notably, neutrophils themselves contribute to the switch to healing, secreting IL‐22 and “restoring order” at mucosal barriers (Denning and Parkos 2013; Zindl et al. 2013). Complimenting the whole switch away from inflammation, IL‐22 also encourages the repolarization of pro‐inflammatory M1 macrophages into anti‐inflammatory M2 macrophages aligned with healing (Kim et al. 2019).

Both IL‐17 and IL‐22 can have pathological effects when over‐ or under‐expressed. When IL‐22 is excessive, the cytokine does not support but impairs regeneration of the epithelial layer, the situation suspected in inflammatory bowel disease (Klotskova et al. 2024). Broquet et al. (2017) also describe “pathogenic epithelial‐destructive inflammation” when IL‐22 is not correctly regulated, recruiting neutrophils and stimulating destructive matrix metalloproteinase release, while dysregulated IL‐17 has been connected with autoimmune diseases and inflammation‐related damages to mucosa (Guglani and Khader 2010; Mills 2023). T_H_17 and T_H_22 cells are depleted in HIV infection, both “highly positive” for HIV co‐receptor CCR5, making them “direct targets for viral infection,” leading to reductions in IL‐17 and IL‐22 expression and severely compromised mucosal protections (Sankaran et al. 2008; Xu et al. 2014). On the other hand, “chronically elevated” IL‐17 and IL‐22 expression is a suspected cancer risk (Kempski et al. 2017). Excessive chronic expression of T_H_17 cells and IL‐17 is also observed in chronic rhinosinusitis, a “severe inflammation of the sinus mucosa” causing mucus and pathogen accumulations in the nose and sinuses (Ramezanpour et al. 2016). In allergic rhinitis, Ramezanpour et al. (2016) note a positive correlation between IL‐17 serum levels and disease severity. Any “failure to terminate” pro‐inflammatory T_H_17 expression risks pathologic states of chronic inflammation (Kempski et al. 2017). Zenewicz (2018) emphasizes that in acute inflammation IL‐22 is protective, but when drawn out in chronic inflammation IL‐22 becomes pathogenic, because of the excessive epithelial proliferation and reduced apoptosis, producing pathologic hyperplasia (atypically high cell numbers). This bad outcome is IL‐22's natural tendencies run amok since epithelial cell proliferation, the cytokine's expertise, is needed for repair and maintenance of mucosal barriers (Chen and Yao 2023). In the balance of immunity, these relationships suggest that as long as chronic inflammation is maintained by type 3/M1/T_H_17 immunity, with IL‐17 expression chronically heightened and IL‐22 dysregulated, the switch to type 4/M2/T_H_22 will be compromised, not only interfering with repairs and maintenance, but also barrier function and integrity.

Returning to the lived context of stressors impacting bodies and barrier function, tobacco smoking and alcohol consumption can have potent impacts on mucosal barriers. Cigarette smoking, for example, leads to neutrophilic inflammation and compromises mucosal barriers, the respiratory mucosa induced into “excessive” mucin production (“mucus hypersecretion”), itself the result of goblet cell metaplasia and hyperplasia (undue increases in goblet cell numbers) in response to the “abundant production” of reactive oxygen species and destructive protease enzymes by neutrophils (Shen et al. 2018). Smoking's impact on goblet cells becomes even more fixed in the neutrophilic inflammation of chronic obstructive pulmonary disease (and chronic bronchitis), also defined by the release of high concentrations of tissue‐damaging neutrophil elastase enzyme and reactive oxygen species with impaired mucociliary clearance in the lung mucosa, causing ciliary injuries and an impaired function that stimulates goblet cell hyperplasia and ongoing mucus hypersecretion, further impairing lung function (Hauber et al. 2006; Wang, Zhang, et al. 2023). Shen et al. (2018) suggest nearly half of COPD patients experience airways clogged with mucus, reducing airflow and furthering disease pathogenesis. In addition to the damage caused by the “chronic infiltration” of neutrophils, COPD is also characterized by a “deficiency” of protective IgA antibodies (Hauber et al. 2006; Wang, Zhang, et al. 2023). Herrero‐Cervera et al. (2022) describe how pro‐inflammatory neutrophil proteases cleave IL‐22 receptors and disrupt that signaling pathway. Meaning that, just as neutrophils are damaging the lungs, they are also inhibiting the type 4/M2/T_H_22 switch that could repair those damages.

Alcohol (ethanol) consumption is another common, regular, or chronic stressor compromising mucosal barriers. Upon consumption, ethanol passes through the mucosal barriers into the bloodstream and then onto the liver and pancreas. Ethanol metabolization in the liver and pancreas causes inflammation (Lafdil et al. 2010), with neutrophil elastase‐ and reactive oxygen species‐expressing neutrophils responding to ethanol's toxic metabolites, causing tissue damage and provoking more inflammation (Szabo et al. 2007). In the balance of immunity, as long as the ethanol stressor remains present and sustaining type 3/M1/T_H_17 inflammation in organs like the liver and pancreas, mucosal barriers (respiratory, digestive, urogenital) will be challenged to initiate periodic type 4/M2/T_H_22 switches for repair and maintenance and, without those checks, will become increasingly more dysregulated. In digestive tract mucosa, according to Llorente and Karin (2025) and Szabo et al. (2007), an “intestinal permeability” emerges with chronic alcohol consumption, caused by disruptions in the epithelium's tight junctions and poor mucosal barrier function. Further to the permeability, however, there is also an overall weakening of barrier defenses described by Llorente and Karin (2025), leading to impaired sIgA production and reductions in innate lymphoid cells and IL‐22 signaling, as well as alterations to dendritic cells and macrophages, altered T cell responses, and an increase in mucin secretion (so much that antimicrobial peptides may be unable to make contact with pathogens).

Via these ethanol‐induced chronic pro‐inflammatory effects, bacteria, viruses, and fungi (whether exogenous pathogens or in the microbiome) may colonize or translocate digestive, respiratory, or urogenital mucosal barriers, accessing the bloodstream and tissues. Alcohol consumption and the type 3/M1/T_H_17 inflammation it produces is a known risk for *Candida albicans* overgrowth (due to poor mucosal controls), Chu et al. (2020) describing increased fecal levels of this fungal member of the microbiome in patients with alcoholic hepatitis. Under conditions of inflammation‐driven mucosal dysregulation, many barrier protections, including those supplied by zymogen granule protein 16 (ZG16), can be compromised. Along with mucin, ZG16 is a “sentinel” protein secreted by goblet cells which binds with the mannan and peptidoglycan in the cell walls of fungi and Gram‐positive bacteria, respectively (Song et al. 2023). ZG16 binds *Candida albicans* , keeping the microbiome‐associated fungus localized to the mucus gel layer, preventing translocation and pathogenic infection (Song et al. 2023), though any mucus disruptions can compromise this protein's important function (Chen et al. 2024). Bacher et al. (2019) describe how dysregulated T_H_17 responses are understood to compromise healthy relationships with *Candida albicans* at mucosal barriers, producing complications like mucocutaneous candidiasis infection. Important fungal protections will be impacted if T_H_22 and IL‐22 secretion are repressed, Zenewicz (2018) noting the “susceptibility” of patients treated with anti‐IL‐22 therapeutics to fungal infection, including chronic mucocutaneous *Candida* infections.

Alcohol consumption and chronic type 3/M1/T_H_17 inflammation also alters barrier protections in the respiratory tract, producing dysregulated, hyper‐inflammatory macrophages (Lewis et al. 2023) and reduced concentrations of neutrophils (Parlet et al. 2015). Trevejo‐Nunez et al. (2015) examined respiratory mucosa responses to *Streptococcus pneumoniae* infection in ethanol‐fed rhesus macaques, observing dysregulated barrier dynamics with downregulation of T_H_17‐related genes and downstream reductions in IL‐17 and IL‐17‐mediated chemokine release which impaired neutrophil recruitment and reduced *Streptococcus pneumoniae* clearance. Ethanol‐fed mice exposed to *Aspergillus fumigatus* (a “ubiquitous fungus” regularly cleared by neutrophils in the airways) also experienced increased fungal burdens and mortality due to reduced IL‐17‐related CXCR2 chemokine signaling for neutrophil recruitment, leading to reduced neutrophil activation, less phagocytosis, and “defective” reactive oxygen species production, all compromising the “killing functions of neutrophils” and allowing for fungal colonization instead of being cleared, producing a “sepsis‐like” response to *Aspergillus* infection and “substantial fungal burdens” (de Oliveira Malacco et al. 2020). Zeng et al. (2023) argue that because of the alcohol‐associated permeability and dysfunction of the intestinal barrier, T_H_17 cells upregulated by the presence and overgrowth of *Candida albicans* (the “strongest known inducer” of T_H_17 cells in humans) subsequently travel by blood to other sites, including the lungs, where those T_H_17 cells primed by a fungus cross‐react with any other fungal encounters, causing chronic lung inflammation because of ongoing inhalation of common fungi like *Aspergillus fumigatus* . Regular (and binge) alcohol consumption is a contemporary concern in the modern population, but the scope hits significantly hard in Indigenous communities where alcohol combined with the everyday, household‐related fungal exposures can produce chronic, dysregulated lung inflammation. In Nunavik, regular or heavy alcohol consumption originally borne out of the traumas of colonization not only disrupts community life, it also weakens mucosal barriers in a context where barrier‐threatening exposures are ever present, chronic and engrained.

Severe asthma risk lies at the intersection of inflammation‐inducing stressors, IL‐17, T_H_17 cells, and neutrophils in the sensitive respiratory mucosa. While there are different types and subtypes of asthma, neutrophilic asthma is a severe asthma phenotype characterized by “neutrophil influx, airway injury, and inflammation” and “pathogenic” T_H_17 cell function (Guo et al. 2025; Mills 2023). Within the respiratory tract, because of sustained and ultimately destructive levels of protease enzymes (matrix metalloproteinases and neutrophil elastase), reactive oxygen species, and pro‐inflammatory cytokines (IL‐17, IL‐1β, TNF‐α) caused by neutrophils, the tissue damage, goblet cell metaplasia, excessive mucus secretion, tissue‐damaging NETs, and hyperresponsive airway produce asthma (Guo et al. 2025). As a result, the myriad chronic stressors producing chronic type 3/M1/T_H_17 inflammation (from anxiety and diabetes, to fungi and endotoxins) can drive the neutrophilic inflammation and dysfunction that triggers severe asthma, likely more readily among those who, by virtue of natural human variation, have strong neutrophil response tendencies or those whose neutrophils are natural heavy oncostatin M (OSM) cytokine producers. Levels of OSM are elevated in neutrophilic asthma patients, a cytokine capable of causing “barrier dysfunction” due to loosened tight junctions and a greater epithelial permeability that allows allergens, pathogens, and antigens to translocate the epithelia, producing onsets of adaptive immunity‐fueled inflammation (Pothoven et al. 2017). Perhaps, breathing in fungal spores encountered in homes, because of these dynamics, some bodies will be naturally more prone to strong type 3/M1/T_H_17 immunity switches, or tend towards higher levels of IL‐17 expression or drive greater neutrophilic recruitment that tips the balance into a strong allergic response, particularly if those instigating fungal exposures are chronic.

Connections between fungal exposures and type 3/M1/T_H_17 inflammation and severe asthma have been traced. Zhang et al. (2017) describe the “immunomodulatory” effects of fungi, specifically the dectin‐1 receptor on dendritic cells that bind with fungal β‐glucans and guide the polarization of T_H_17 cells (alongside T_H_2 cells), producing the type 3/M1/T_H_17 inflammation that triggers severe asthma. Likewise, Xie et al. (2022) target the role of T_H_17 cells in “neutrophilic” (in comparison to T_H_2 asthma's eosinophilic phenotype) airway inflammation and severe asthma, the overexpression of IL‐17 inducing the chemokines which draw the influx of neutrophils, the key suspects responsible for the “enhanced severity” of fungi‐associated asthma (van Tilburg Bernardes et al. 2020). While Xie et al. (2022) explore the (type 3/M1/T_H_17) inflammation associated with smoking and obesity promoting and worsening neutrophilic asthma, to this list could be added all of the other chronic exposures inducing type 3/M1/T_H_17 inflammation and contributing to the “excessive T_H_17 cell responses” associated with neutrophilic asthma. Rennie et al. (2020) assessed risk factors for allergic and non‐allergic asthma in First Nations children in Saskatchewan, their regression analyses identifying a significant connection between childhood allergic asthma and homes with damage due to dampness, homes with signs of mildew/mold, and obesity. All three of these risk factors for severe asthma connect with chronic type 3/M1/T_H_17 immune switches and, because of an ongoing tide of T_H_17 signaling, drive the excessive recruitment and damaging impacts of neutrophils.

Extending the perspective further, along with asthma, chronic type 3/M1/T_H_17 inflammation switches driven by chronic fungus and endotoxin exposures, particularly with mucosal barriers compromised by alcohol and tobacco smoking, are a risk for barrier translocating and potentially invasive bacterial, viral, and fungal infections, involving either microbiome members or acquired species. Bacher et al. (2019) identify dysregulated T_H_17 immunity as a risk for *Staphylococcus aureus* infection, a common resident of the microbiome which can become opportunistically pathogenic with great invasive potential. In their review, Brinkworth and Shaw (2022) connect invasive sepsis‐causing infections with chronically upregulated pro‐inflammatory cytokines. In their argument, sepsis caused by chronic stress and inflammation becomes a real and tangible “immunological embodiment” of colonialism and racism. Notably, as Brinkworth and Shaw argue, “racialized and marginalized people get and die of sepsis more frequently than heirs of colonial power.” Acknowledging “insidious, systemic factors cause racial disparities in sepsis,” Brinkworth and Shaw (2022) argue the evidence that sepsis‐risking stressors can be reduced by government action, particularly in housing improvements and immunization programs. Like Brinkworth and Shaw (2022), this paper is also grounded in the pro‐inflammatory immunological toll of colonization and also focuses on the issue of inadequate housing, particularly how chronic type 3/M1/T_H_17 inflammation induced by chronic everyday exposures to fungi and endotoxins compromise type 4/M2/T_H_22 switches and produce vulnerable and dysregulated mucosal barriers, enabling pathogenic microbial translocations. Through these mechanisms, the stressors of colonialism effectively weaken protective mucosal barriers and heighten infection risks.

Brinkworth and Shaw (2022) undertake a deep analysis connecting stressors with colonial legacies and ongoing, inflated sepsis risks in global Indigenous populations. Huang et al. (2021) describe invasive bacterial diseases as “elevated among the Indigenous peoples in countries with Arctic regions,” including a “high burden of disease in Canada's northern populations.” The annualized incidence rate (per 100 000) for invasive pneumococcal disease (IPD) is 31.3 and 7.0 among Indigenous and non‐Indigenous peoples, respectively, 14.8 versus 2.7 for invasive group A streptococcus (iGAS), and 13.1 versus 0.9 for Hi ( *Haemophilus influenzae* ), all statistically significant Indigenous/non‐Indigenous differences in Canada (Huang et al. 2021). In Ontario's Northwestern Health Unit, which covers one‐fifth of the province and 39 First Nations, the rate of invasive pneumococcal disease is estimated to be three times higher than the provincial rate (NWHU 2025). Invasive infections produce atypical effects, with *Streptococcus pyogenes* , the bacterium connected with scarlet fever and pharyngitis, able to cause necrotizing fasciitis or cellulitis when the infection becomes established in typically sterile sites (Mercadante et al. 2024).

Kirlew et al. (2014) identify diabetes and skin disease as notable comorbidities of community‐acquired methicillin resistant *Staphylococcus aureus* (CA‐MRSA), the same comorbidities Bocking et al. (2016) connect with invasive group A streptococcus (iGAS), along with regular alcohol consumption, in a study of 26 rural and remote First Nations in Northwestern Ontario. Collectively, diabetes, skin disease, and alcohol all engage type 3/M1/T_H_17 pro‐inflammatory switches. The role of colonization in these comorbidities, the connections between diabetes, alcohol, multigenerational trauma, assimilation, and racism, cannot be understated, the incidence of iGAS in Northwestern Ontario's First Nations is 56.2 per 100 000, “more than 10 times higher” than rates for Canada and Ontario (4.72 and 4.6 per 100 000, respectively), higher also than Li et al.'s (2016) estimates of iGAS incidence among Indigenous peoples in Northern Canada, which Bocking et al. (2016) suspect is because of a greater diabetes prevalence in First Nations (which draws chronic type 3/M1/T_H_17 inflammation and dysregulates mucosal protections). Analyzing data for 2006–2013, Li et al. (2016) found the incidence of invasive pneumococcal disease, invasive group A streptococcus, and *Haemophilus influenzae* was 2.6 to 10 times higher in Northern Canada, particularly among First Nations and Inuit infants and seniors, relative to the rest of Canada. In Alberta, for 2017, Tyrrell et al. (2021) estimated iGAS rates at 52.2 cases/100000, a clear “6‐fold higher” in comparison to 8.7/100000 in non‐First Nations persons, with the most prevalent iGAS risk factors in First Nations being chronic pro‐inflammatory (type 3/M1/T_H_17) switch‐inducing (and type 4/M2/T_H_22 mucosal barrier compromising) diabetes, hepatitis C, and alcohol and drug dependencies, and with transmission facilitated by overcrowding (Tyrrell et al. 2021).

In their study of invasive *Haemophilus influenzae* in Northwestern Ontario, Brown et al. (2009) found Indigenous patients disproportionately affected, accounting for 53% of cases (20 of 38 cases) over 7 years, even though Indigenous peoples constitute only 20% of the population of Northwestern Ontario. In terms of *Haemophilus influenzae* comorbidities, invasive risk is heightened in children under 5 and among individuals with diabetes, COPD, chronic renal failure, and Crohn's disease, all described as “compromising the immune system” (Brown et al. 2009). Other than the children (whose immune systems are still developing), the compromise really comes in ongoing, chronic type 3/M1/T_H_17 pro‐inflammatory switches occurring at the expense of regular, healthy type 4/M2/T_H_22 switches, dysregulating the respiratory mucosa. Likewise, Torres et al. (2015) identify COPD, asthma, smoking, diabetes, and chronic heart disease (including congestive heart failure, and cardiovascular and valve diseases) as invasive pneumococcal disease risks, all engaging chronic type 3/M1/T_H_17 inflammation which dysregulate the respiratory mucosa and sacrifice repair and maintenance. It is no surprise that comorbidities for invasive infections overlap with comorbidities for latent Mtb reactivation, the disease potential of chronic type 3/M1/T_H_17 inflammation compromising both types of anti‐inflammatory immune switches, the type 4/M2/T_H_22 barrier protections and the type 2/M2/T_H_2 switch which keeps Mtb latent in granulomas.

The connections between chronic heart failure and opportunistic pneumococcal pneumonia infections have long been known, Musher et al. (2007) describing how early 20th century physicians “routinely prescribed digitalis for pneumonia and penicillin for chronic heart failure.” Among patients hospitalized for pneumococcal pneumonia between 2001 and 2005, Musher et al. (2007) identified “substantial risk for a concurrent acute cardiac event,” including myocardial infarction, serious arrhythmia, and new or worsening chronic heart failure, at the time of admission. Cardiac complications drive pro‐inflammatory cytokine signaling (Torres et al. 2015) of the type 3/M1/T_H_17 kind, stealing the switch away from type 4/M2/T_H_22 immunity and creating opportunities for respiratory barrier‐translocating pneumonia infections and mortality complications, particularly since the pneumonia infection stresses the heart further, increasing oxygen demand just as oxygenation is “compromised,” potentially disrupting atherosclerotic plaques, promoting blood clots, and suppressing ventricular function (Musher et al. 2007). Adding to the scope for underlying chronic type 3/M1/T_H_17 inflammation, Musher et al. (2007) describe their study population as also possessing “a high prevalence of tobacco abuse.” In their study of “survivors of hospitalization for community‐acquired pneumonia,” Yende et al. (2008) identified those with the highest serum IL‐6 (a pro‐inflammatory cytokine) concentrations at discharge at increased risk of dying within 3 months due to cardiovascular events, repeat infections, renal failure, or cancer. Significantly, Yende et al. (2008) describe that “circulating IL‐6 concentrations were 10‐ to 15‐fold lower than concentrations seen during severe sepsis, but threefold higher than concentrations in older adults at risk for community‐acquired pneumonia,” clearly identifying the scope of ongoing type 3/M1/T_H_17 inflammation, immunity out of equilibrium at the expense of type 4/M2/T_H_22 mucosal barrier‐protective switches.

Blastomycosis is a fungal (*Blastomyces dermatitidis*) infection that can occur with environmental exposures to fungal spores growing in decomposing wood and leaves in moist or wet soil (Xie 2025). When breathed in, microscopic spores enter the lungs, where, Xie (2025) describes, human body temperature transforms the spores into yeast. While about half of infections are asymptomatic or mild, in other cases, Xie (2025) noting those with “impaired immune systems,” severe pulmonary infection or extrapulmonary disease can develop as the fungal infection translocates the respiratory mucosa and disseminates via the bloodstream. Expanding the idea of “impaired,” this paper suggests the impairment arises under chronic type 3/M1/T_H_17 inflammation interfering with type 4/M2/T_H_22 switches maintaining healthy respiratory mucosa that would better resist blastomycosis infections. Between November 2021 and January 2022, 50 cases of blastomycosis and 5 blastomycosis‐related deaths occurred in Constance Lake First Nation, a community of about 800 in northern Ontario. A Coroner's Inquest was undertaken, the jury returning 79 recommendations, including one calling for “comprehensive inspections of houses” in Constance Lake, specifically because of community concerns over mold (Rutherford 2025). Rutherford (2025) notes, “the inquest heard that people in Constance Lake were concerned that sewage back‐up in their basements created mold that contributed to the respiratory illness.” According to immune balances, alleviating any chronic type 3/M1/T_H_17 inflammation‐inducing mold exposures would help preserve periodic type 4/M2/T_H_22 immune switches that maintain healthy respiratory mucosa. By lightening the immunological toll of chronic inflammatory stressors like mold, well‐maintained and functioning mucosa would support greater resistance to environmental *Blastomyces* exposures.

## 7 Conclusion

In the context of tuberculosis, the nature of communities and their particular niche‐based stressors, paired with the scope of human immunological variation and variable bacterial virulence factors shape latent Mtb reactivation risks. Latent Mtb reactivation risks typically have less to do with “immunosuppression” and much more to do with type 1/M1/T_H_1 and type 3/M1/T_H_17 immunity switches, pro‐inflammatory states which Mtb can use to escape their granulomas and cause disease. Returning to Nunavik's high incidence rate, various niche‐based stressors, from tobacco smoking and alcohol consumption, to COPD, anxiety, and chronic toxoplasmosis can drive chronic or regular type 3/M1/T_H_17 switches. In Nunavik, and in other Indigenous communities in Canada, housing can also be the source of chronic potent type 3/M1/T_H_17 inflammation, not only in connection with chronic fungal and endotoxin exposures, but also because of anxieties and tensions that can arise with overcrowding. In addition to heightening reactivation risks in populations carrying high latent Mtb burdens, this paper suggests housing's pro‐inflammatory stressors also drive immunological connections to severe asthma, and bacterial, viral, and fungal colonizations or translocations of respiratory, digestive, or urogenital mucosal barriers, leading to infections, some with invasive potential.

Medically treating cases of Mtb reactivation in an effort to stem an epidemic outbreak is important, but cannot be the only approach to manage more broadly the ongoing reactivation risks emerging in pro‐inflammatory contexts shaped by ongoing legacies of settler colonialism. As Coussens et al. (2017) argue, a broader approach engaging public health and a sensitivity to “the adverse environmental and social exposures associated with reactivation” would not only reduce tuberculosis risks, but also reshape risks for a wealth of other diseases. In Canada, addressing the housing issue in Indigenous communities would help reduce risks not only for latent Mtb reactivation and ongoing transmission, but also severe asthma and infections ranging from respiratory syncytial virus to necrotizing fasciitis and severe blastomycosis. As Coussens et al. (2017) suggest, “if we were to address the social conditions that foster TB reactivation, then there would be little need to mass‐produce and distribute community‐wide preventive therapy,” a therapy which would, notably, be addressing only one disease. Very significantly, Coussens et al. (2017) stress population groups “are not inherently vulnerable” to Mtb reactivations “but are chronically exposed to adverse conditions that place them in an at‐risk category.” Likewise, reflecting on the use of vaccines to reduce iGAS risk in the midst of comorbidities like obesity and diabetes, Williamson et al. (2016) argue, “these interventions are at the ‘fire‐fighting’ end of the public health spectrum,” since “concerted efforts should be made to tackle the obesity and diabetes tsunami,” tapping into the true inflammatory roots of disease.

Medicine and research are evolving therapies to manage dysfunctioning bodies, calling for IL‐22 therapies to offset the effects of T_H_17‐driven inflammation (Chen and Yao 2023), for example, or employing a CXCR2 blocking antibody in attempts to curb the destructive excess of neutrophils in COPD (Herrero‐Cervera et al. 2022). While these treatments will undoubtedly help to alleviate suffering, they are more examples of “fire‐fighting,” which would ideally pair with more ‘on the ground’ efforts to reduce the root causes of type 3/M1/T_H_17 inflammation, particularly the chronic kind that has come to increasingly impact modern human life. This paper argues that “adverse conditions” driving acute and chronic inflammation can be population‐specific, niche‐based stressors that induce, most often, disruptive and chronic type 3/M1/T_H_17 inflammation, emphasizing the need to tackle those stressors head on, including the housing crisis, rather than focus the weight of the effort on the diseases they produce. Improving housing is a fundamental first step, Minich et al. (2011) arguing its ability to “positively influence conditions that lead to thriving Indigenous health,” narrowing the clear health disparities that continue to exist between Indigenous and non‐Indigenous peoples in Canada.

## Funding

The author has nothing to report.

## Ethics Statement

The author has nothing to report.

## Consent

The author has nothing to report.

## Conflicts of Interest

The author declares no conflicts of interest.

## Data Availability

The author has nothing to report.

## Endnotes

^1^
Inuit residents of Nunavik.

^2^
One of three groups of Indigenous Peoples in Canada, along with First Nations and Metis people. Inuit traditional homelands stretch across Arctic and Subarctic regions in Canada, Greenland, Alaska, and Siberia.

^3^
For insights on the history of tuberculosis among Inuit in Canada, including the practice of sending those with active disease to sanatoria and hospitals in the south, “a long way from home,” see Grygier (1994).

^4^
Four Inuit land claim regions comprise Inuit Nunangat, including Inuvialuit Nunangat/Inuvialuit Settlement Region, Nunatsiavut, Nunavik, and Nunavut. The four regions are mapped here: https://itk.ca/wp‐content/uploads/2023/03/2023‐Inuit‐Nunangat‐Map‐Simplified.pdf (on the Inuit Tapiriit Kanatami website https://itk.ca).

^5^
Chronic (ongoing, long‐lasting), ingrained (firmly embedded, difficult to change), niche‐based (an evolutionary grounding, from Dressler's (2019) “culture as an evolutionary niche” and Laland et al.'s (2001) perspetives on “niche construction”). The niche is widely conceptualized, considering communities in their social, cultural, temporal, and environmental context. Niches can be powerfully shaped by culturally‐constructed stressors (such as colonization, industrialization, the synthetic chemical revolution, and capitalism) and continue to evolve generationally.

^6^
Testimonies of witnesses are provided in the documentary “Echo of the Last Howl” (2004, Taqramiut Productions Inc.), available on Makivvik's YouTube channel (https://www.youtube.com/watch?v=IpHPH4VYKAA).

^7^
Royal Canadian Mounted Police.

^8^
APTN National News covered the story, available on the APTN News YouTube channel (25 November 2024): https://www.youtube.com/watch?v=tQEtYhe50kM. In 2011, the Province of Quebec offered an apology for the role of the Quebec provincial police.

^9^
Census data from 2021 indicates an estimated 40% of First Nations people (with Registered or Treaty Indian status) live on‐reserve, meaning they reside on "land designated by the federal government to be used and occupied by First Nations communities" (Lyeo et al. 2024). Off‐reserve First Nations people live elsewhere.

^10^
Band‐owned homes are owned collectively by the First Nation (Band) and rented from the Band Council (the reserve land itself is owned by the Crown).

^11^
The Band Council is the local self‐governance on a First Nation responsible for managing federal funding and property taxes, administering programs, and representing members who live on‐ and off‐reserve.
